# The presentation of metabolic dysfunction and the relationship with energy output in breast cancer survivors: a cross-sectional study

**DOI:** 10.1186/1475-2891-12-99

**Published:** 2013-07-15

**Authors:** Emer M Guinan, Elizabeth M Connolly, M John Kennedy, Juliette Hussey

**Affiliations:** 1 Discipline of Physiotherapy, School of Medicine, Trinity Centre for Health Sciences, St. James’s Hospital, Dublin 8, Ireland; 2 Department of Surgery, St. James’s Hospital, Dublin, Ireland; 3 Academic Unit of Clinical and Medical Oncology, St. James’s Hospital and Trinity College, Dublin, Ireland

**Keywords:** Breast cancer, Insulin resistance, Metabolic syndrome, Resting metabolic rate, Physical activity, Prognosis

## Abstract

**Background:**

Breast cancer prognosis can be adversely influenced by obesity, physical inactivity and metabolic dysfunction. Interventions aimed at improving surrogate markers of breast cancer risk such as insulin resistance may result in improved breast cancer outcomes. The design of such interventions may be improved through increased understanding of metabolic presentation in this cohort. This cross-sectional study aimed to characterise the metabolic profile of breast cancer survivors relative to abdominal obesity and insulin resistance. A secondary aim was to compare measures of energy output across these groups.

**Methods:**

Sixty-nine women (mean (SD) age 53.43 (9.39) years) who had completed adjuvant chemotherapy and radiotherapy for breast cancer were recruited. All measures were completed during one assessment conducted 3.1 (1.0) years post diagnosis. Body composition was measured by bioimpedance analysis and waist circumference (WC). Fasting (12 hour) blood samples were drawn to measure lipid profile, glucose, insulin, glycosylated haemoglobin A1c (HBA1c) and C-reactive protein (CRP). Insulin resistance was estimated by the homeostatic model assessment index (HOMA-IR)). Energy output was evaluated by resting metabolic rate (RMR) measured by indirect calorimetry and physical activity measured by accelerometry. Characteristics were compared across four groups (1. WC <80 cm, not insulin resistant; 2. WC 80–87.9 cm, not insulin resistant; 3. WC >88 cm, not insulin resistant; 4. WC >80 cm, insulin resistant) using ANOVA (p < 0.05).

**Results:**

Group 4 was characterised by significant disturbances in measures of glucose metabolism (glucose, insulin, HOMA-IR and HBA1c) and raised CRP compared to other groups. Group 4 also displayed evidence of dyslipidemia and higher body composition values compared to Groups 1 and 2. Both absolute and adjusted RMR were significantly higher in the Group 4 versus all other groups. Physical activity levels were similar for all groups.

**Conclusions:**

The results from this study suggest that participants who were both centrally obese and insulin resistant showed evidence of dyslipidemia, low-grade inflammation and glucose dysregulation. Metabolic profiles of participants who were centrally obese only were not significantly different from lean participants. Consideration of baseline metabolic presentation may be useful when considering the therapeutic targets for future interventions in this cohort.

## Introduction

Metabolic dysfunction, including the metabolic syndrome (MetSyn) and insulin resistance, is a long-term complication of curative treatment for many cancers including breast [1], prostate [2] and testicular [2]. There is increasing evidence that metabolic disturbance, characterised by insulin resistance [3,4], altered adipokines [3] and chronic low-grade inflammation [5], is associated with increased breast cancer and all-cause mortality and a higher risk of breast cancer recurrence. Breast cancer survivors represent a unique group who endure several treatment associated alterations in lifestyle habits including weight gain [6] and reduced physical activity levels [7], along with high risk of ovarian failure in premenopausal women [8], leading to early onset menopause and consequential worsening metabolic profiles [9]. However, not all breast cancer survivors are overweight and inactive and not all overweight and obese women are metabolically unhealthy [10]. The presentation of metabolic disturbance varies greatly among obese individuals [11] and there is a need for greater understanding of the characteristics of metabolic dysfunction in breast cancer survivors in order to design targeted weight-loss and exercise interventions.

The classification of metabolically unhealthy has not been standardised due to the ever-evolving definition of the MetSyn and the ongoing debate regarding the inclusion of a measure of insulin resistance in the syndrome definition [12-14]. The current MetSyn definition includes central obesity, elevated fasting glucose, atherogenic dyslipidemia and hypertension [12]. Insulin resistance is a central component of the MetSyn and is arguably one of the most important features of metabolic dysfunction in relation to cancer due to the mitogenic effects of insulin on breast cancer cells [15] and the substantial evidence linking hyperinsulinemia and insulin resistance to cancer prognosis [3]. Equally central obesity, characterised by increased visceral fat accumulation, is considered a core component of the MetSyn and is associated with the secretion of many pro-tumour products including pro-inflammatory cytokines (interleukin (IL)-6 and tumor necrosis factor (TNF)-α), adipokines and sex hormones [16]. There is a lack of consensus regarding the cut-point for central obesity, as measured by waist circumference (WC). Generally, 80 cm is the accepted cut-point for central obesity in women while ≥88 cm is associated with substantially increased disease risk [12]. While most individuals with the MetSyn are also insulin resistant [13], the relationship between insulin resistance and atherogenic dyslipidemia and hypertension is unclear, particularly due to the inclusion of drug therapies for lipid abnormalities and hypertension in the most recent MetSyn definition. In breast cancer survivors, it may be useful to examine metabolic dysfunction in terms of insulin resistance and visceral obesity as these measures may be potentially more predictive of cancer-specific outcome.

Long-term obesity is essentially due to an imbalance between energy intake and energy output. Resting metabolic rate (RMR) is the largest component of energy expenditure [17] and has been shown to be altered in the presence of metabolic abnormalities [18]. Equally habitual physical activity, which represents up to 15% of total energy expenditure, is a key factor in the development and management of the MetSyn [14] and may positively influence breast cancer prognosis [19]. The aim of the current study was to characterise the metabolic profiles of breast cancer relative to worsening central obesity and insulin resistance. A secondary aim was to compare energy output across these groups and to examine associations between energy expenditure and metabolic parameters.

## Methods

### Study design and participants

The study was designed as a cross-sectional study. A convenience sample of breast cancer survivors were recruited from oncology clinics at St. James’s Hospital, Dublin Ireland from May 2011 to June 2012. Participants were eligible for the study if they had completed all adjuvant breast cancer treatment including at least one of the following: chemotherapy, radiotherapy and anti Her-2 directed biological therapy. Those continuing to take adjuvant hormonal therapy were eligible to participate. Evidence of active or recurrent disease, co-morbidities including type II diabetes mellitus (T2DM) or age >70 years were considered exclusion criteria. Ethical approval was granted by the hospital research ethics committee and all participants provided written, informed consent prior to measurements.

### Data collection

Body composition, including body weight, percentage body fat and fat free mass (FFM), was estimated by bioimpedance analysis using the Tanita MC 180 MA Multi-Frequency Body Composition Analyzer (Tanita Corp, Tokyo, Japan). Standing height was measured barefoot to the nearest millimetre using a SECA stadiometer. WC was measured using a flexible measuring tape, in duplicate, to the nearest millimetre, at the midpoint between the top of the iliac crest and the last rib [20]. Blood pressure measurements were taken on the non-surgical side in duplicate using the auscultatory method. Fasting (12 hour) venous blood samples were drawn to measure glucose, insulin, lipid profile (total cholesterol (TC), high-density lipoprotein cholesterol (HDL-C), low-density lipoprotein cholesterol (LDL-C) and triglycerides (TG)), (glycosylated haemoglobin A1c (HBA1c)) and C–Reactive Protein (CRP). Insulin Resistance was calculated using the Homeostatic Model Assessment Index (HOMA-IR): [(fasting glucose (mmol.L^-1^) × fasting insulin (mU.L^-1^)/22.5] [21]. Insulin resistance was defined as the 75^th^ percentile of HOMA-IR of participants studied (HOMA-IR = 2.45) [22]. The MetSyn was diagnosed in the presence of any three of the following: elevated WC (≥80 cm); elevated TG (≥1.7 mmol.L^-1^) or drug therapy for lipid abnormalities; reduced HDL-C (<1.3 mmol.L^-1^) or drug therapy for lipid abnormalities; elevated blood pressure (systolic ≥130 mmHg and/or diastolic ≥85 mmHg) or antihypertensive medication; elevated fasting glucose (≥5.6 mmol.L^-1^) or glucose-lowering medication [12].

Insulin was measured by electrochemiluminescence immunoassay (Elecsys Insulin Assay, Roche Dianostics GMBH). Enzymatic, colorimetric assays (Roche/Hitachi **cobas c** systems) were used to measure fasting glucose, TC, HDL-C and TG. LDL was calculated using the Friedewald equation. High performance liquid chromatography (Arkray/Adams A1c HA-8160 Analyser System) was used to measure HBA1c and CRP was measured by particle enhanced immunoturbidimetric assay (Roche/Hitachi **cobas c** systems). Laboratory analyses were carried out at the national biochemistry reference laboratory at St. James’s Hospital, Dublin.

Energy output was measured by RMR and habitual physical activity. RMR and respiratory quotient (RQ) were measured by indirect calorimetry using the COSMED Metabolic Quart according to the guidelines of Compher and colleagues [23]. Measurements were completed with the participant in the supine position following a 10–20 minute rest period. Respiratory gas exchange was measured continuously for 20 minutes, excluding the first five minutes of data, using a canopy collection device. Data was obtained from at least 5 minutes of a stable measurement (coefficient of variation <10% (variation in oxygen consumption (VO2) ≤10%; variation in carbon dioxide production (VCO2) ≤10%). RMR was expressed in absolute values (kJ.day^-1^) and as RMR adjusted for FFM (kJ.day^-1^.kg^-1^) [24]. Physical activity was measured objectively using the RT3 activity monitor (Stayhealthy Inc Montrovia, CA, USA). The RT3 is a valid [25], reliable [26] tool and was used to quantify percentage time spent in each domain of activity and to monitor adherence to physical activity guidelines (≥30 minutes of moderate intensity activity, accumulated in ≥10 minute bouts, five days per week [27]). Activity intensity was defined using validated cut-points [25] and sedentary behaviour was defined as an activity count ≤100 [28]. Habitual activity patterns in the preceding year were measured subjectively using the Minnesota Leisure Time Physical Activity Questionnaire (MLTPAQ). The MLTPAQ is a valid [29] interview-administered questionnaire designed to measure the frequency of leisure-time and recreational activities in the preceding year and combines this information with intensity scores to measure physical activity in terms of activity metabolic index (AMI) and expressed as metabolic equivalent (MET)-minutes per day (MET-min.day^-1^).

Data was analysed using SPSS version 18.0 software (SPSS, Inc, Evanstron, IL). Distributions were checked for normality using the K-S test and log-transformations were applied to non-normally distributed data. Data are presented as mean ± standard deviation. Participants were classed into four subgroups: Group 1: healthy WC (<80 cm) and not insulin resistant; Group 2: elevated WC (WC 80–87.9 cm) and were not insulin resistant; Group 3: substantially increased waist circumference (WC >88 cm) and not insulin resistant; and Group 4: both centrally obese and insulin resistant. With the exception of one participant who had a waist circumference of 82.4 cm, all women in Group 4 had a WC >88 cm. Anthropometric, metabolic and energy output variables were compared between the four groups using analysis of variance (ANOVA). Significance was set at p < 0.05. Post hoc analysis was completed using Tukey’s HSD method when a significant group effect was observed. Kruskal-Wallis non-parametric ANOVA was used to compare distributions between non-parametric data (BMI, glucose, HBA1c and CRP) and post hoc analysis was completed using Mann–Whitney U test for comparison between pairs of group Corrections were made for multiple comparisons using the bonferroni correction (p < 0.01). Correlations between energy output and metabolic variables were examined using Pearson Product Moment Correlation Coefficients (PPMCC) for normally distributed data and Spearman’s rho for non-normally distributed data. The lowest detectable value for CRP was 1 mg.L^-1^ and therefore results <1 mg.L^-1^ were assigned a value of 1 mg.L^-1^. Values >15 mg.L^-1^ were excluded these most likely represented an acute inflammatory event [30].

## Results

Sixty nine participants (mean (SD) age 53.43 (9.39)) entered the study. The mean time since diagnosis for the overall group was 3.1 (1.0) years. Participant demographics and treatment details are shown in Table 1. Groups were similar for all demographics (p > 0.05). The majority of participants were treated with adjuvant chemotherapy using standard anthracycline and taxane based regimens. Almost 80% of the group were taking adjuvant hormonal therapy.

**Table 1:** Participant demographics

**Characteristic**	**Group 1 (n = 19)**	**Group 2 (n = 16)**	**Group 3 (n = 16)**	**Group 4 (n = 18)**
**Participant demographics**				
**Age (years)**	52.04 (11.7)	50.27 (8.48)	55.65 (9.49)	55.59 (6.69)
**Time since diagnosis (years)**	3.39 (0.90)	2.69 (0.92)	3.19 (0.85)	3.14 (1.22)
Menopausal status*				
Premenopausal	11 (58%)	11 (69%)	8 (50%)	8 (44%)
Postmenopausal	8 (42%)	5 (31%)	8 (50%)	10 (56%)
**Marital status**				
Married	16 (84%)	10 (63%)	11 (69%)	11 (61%)
Single	3 (16%)	6 (37%)	5 (31%)	5 (39%)
**Smoking history**				
Current smoker	1 (5%)	9 (56%)	2 (13%)	6 (33%)
**Cancer details**				
**Cancer stage**				
I	6 (32%)	4 (25%)	5 (31%)	4 (22%)
II	9 (47%)	9 (56%)	8 (50%)	10 (56%)
III	4 (21%)	3 (19%)	3 (19%)	4 (22%)
**Estrogen receptor positive**	14 (74%)	14 (88%)	14 (88%)	14 (78%)
**Surgery**				
Wide local excision	9 (47%)	5 (31%)	10 (63%)	7 (39%)
Mastectomy	10 (53%)	11 (69%)	6 (38%)	11 (61%)
Axillary clearance	7 (37%)	9 (38%)	4 (25%)	10 (56%)
**Chemotherapy**	17 (90)	14 (88%)	13 (81%)	16 (89%)
**Radiotherapy**	15 (79)	11 (69%)	14 (88%)	16 (89%)
**Hormone therapy**				
Tamoxifen	9 (47%)	7 (44%)	12 (75%)	7 (39%)
Aromatase inhibitor	5 (26%)	6 (38%)	2 (13%)	7 (39%)
**Biological therapy**				
Herceptin	3 (16%)	2 (13%)	1 (6%)	2 (11%)

Data are presented as mean (standard deviation) for continuous variables and as frequency (percentage) for categorical variables.

*Refers to menopausal status at diagnosis.

Anthropometric and metabolic parameters are shown in Table 2. Women in Group 3 and Group 4 demonstrated significantly higher anthropometric measures, characterised by significantly greater body weight (p < 0.001 vs. Group 1 and p < 0.002 vs. Group 2), BMI (p < 0.001 for both), WC (p < 0.001 for both) and percentage body fat (p < 0.001 for both) when compared to Group 1 and Group 2. FFM was significantly higher in both Group 3 (p = 0.002) and Group 4 (p = 0.001) compared to Group 1. Metabolic profiles were significantly worse in Group 4 than other groups characterised by significantly lower HDL-C (Group 4 vs. Group 1 p = 0.02; vs. Group 2 p = 0.008), elevated TG (Group 4 vs. Group 1 p < 0.001; vs. Group 2 p = 0.009), measures of insulin-glucose metabolism (fasting glucose, fasting insulin, HOMA-IR and HBA1c, p < 0.001 vs. all groups) and raised CRP (Group 4 vs. Group 1 p < 0.001; vs. Group 2 p = 0.005; vs. Group 3 p = 0.01). Metabolic profiles did not vary between non-insulin-resistant groups.

**Table 2:** Comparison of anthropometric and metabolic variables across groups

**Characteristic**	**Group 1 (n = 19)**	**Group 2 (n = 16)**	**Group 3 (n = 16)**	**Group 4 (n = 18)**
Age (years)	52.16 (11.69)	50.37 (8.52)	55.56 (9.42)	55.61 (6.77)
**Anthropometry**				
Body weight (kg)	59.73 (6.76)	66.22 (5.68)	79.55 (12.58)^a, b^	79.61 (13.85)^a, b^
BMI (kg.m^-2^)	23.09 (21.7-24.6)	24.86 (23.5-26.6)^a^	30.21 (27.5-32.0)^a, b^	30.84 (26.0-36.3)^a, b^
*BMI categories*				
Healthy weight (18.5-24.9 kg.m^-2^)	17 (90%)	8 (50%)	0	1 (6%)
Overweight (25–29.9 kg.m^-2^)	2 (10%)	8 (50%)	10 (62%)	8 (44%)
Obese (> 30 kg.m^-2^)	0	0	6 (38%)	9 (50%)
Waist circumference (cm)	74.05 (4.65)	83.20 (2.54)^a^	95.18 (6.56)^a, b^	97.45 (10.57)^a, b^
Percentage body fat (%)	31.15 (3.69)	33.41 (3.08)	40.37 (3.04)^a, b^	40.19 (5.21)^a, b^
Fat free mass (kg)	40.978 (3.59)	44.08 (4.19)	47.22 (5.77)^a^	47.12 (5.37)^a^
**Blood pressure**				
Systolic blood pressure (mmHg)	114.74 (15.57)	117.00 (9.39)	119.09 (9.98)	125.92 (13.49)
Diastolic blood pressure (mmHg)	77.29 (9.96)	75.69 (4.79)	78.50 (8.73)	80.56 (7.91)
Hypertensive (BP >130/85 mmHg)	4 (21%)	2 (13%)	5 (31%)	9 (50%)
Anti-hypertensive medication	2 (10%)	2 (13%)	4 (25%)	6 (33%)
**Lipid profile**				
TC (mmol.L^-1^)	5.24 (0.98)	5.33 (1.09)	4.61 (0.53)	5.33 (0.93)
Dyslipidemia (TC > 5.2 mmol.L^-1^)	9 (47)	9 (56)	3 (19)	9 (50)
HDLC (mmol.L^-1^)	1.83 (0.41)	1.89 (0.51)	1.62 (0.36)	1.40 (0.41)^a, b^
LDLC (mmol.L^-1^)	2.98 (0.78)	3.13 (1.32)	2.38 (0.67)	3.11 (0.89)
TG (mmol.L^-1^)	0.92 (0.68-1.09)	1.07 (0.7-1.4)	1.16 (0.8-1.6)	1.46 (1.0-1.8)^a, b^
Cholesterol medications	0	1 (6%)	3 (19%)	0
**Glucose metabolism**				
Fasting glucose (mg.dL^-1^)	4.72 (4.3-5.0)	4.81 (4.7-5.0)	4.86 (4.6-5.2)	5.32 (4.8-5.5)^a, b, c^
Fasting insulin (mU.L^-1^)	6.73 (5.5-6.9)	7.30 (5.7-9.8)	8.38 (7.3-9.6)	14.49 (11.6-19.4)^a, b, c^
Hyperinsulinemia (>12 mU.L^-1^)	0	0	1 (6%)	14 (78%)
Insulin resistance (HOMA-IR)	1.42 (1.1-1.8)	1.61 (1.2-2.1)	1.81 (1.4-2.1)	3.43 (2.5-4.2)^a, b, c^
HBA1c (mmol.mol^-1^)	35.21 (33.0-38.0)	35.25 (33.5-37.0)	35.75 (34.0-37.0)	38.39 (33.5-41.0)^a, b^
Glucose medications	0	0	0	0
**Inflammatory marker**				
CRP (mg.L^-1^)	1.53 (1.0-1.8)	1.89 (1.0-2.2)	2.09 (1.1-2.4)	4.98 (1.4-7.0)^a, b, c^
**Metabolic syndrome**	0	3 (19%)	5 (31%)	12 (67%)

Data are displayed as mean (± standard deviation) for normally distributed variables and median (inter-quartile range) for non-normally distributed variables and as frequency (percentage) for categorical variables. Abbreviations: *kg* kilogram, *BMI* body mass index, *TC* total cholesterol, *HDL-C* high density lipoprotein cholesterol, *LDL-C* low density lipoprotein cholesterol, *TG* triglycerides, *HOMA-IR* homeostatic model assessment index, *HBA1c* glycosylated haemoglobin A1c, *CRP* C - reactive protein.

a = significantly different from Group 1; b = significantly different from Group 2; c = significantly different from Group 3.

Energy output variables across the four groups are presented in Table 3. Absolute RMR (kJ.day^-1^) was significantly higher in Group 4 compared to all other groups (vs. Group 1 p < 0.001, vs. Group 2 p = 0.005 and vs. Group 3 p = 0.05). Results remained significant when RMR was adjusted for FFM (kJ.day^-1^.kg^-1^) (Group 4 vs. Group 1 p < 0.001; vs. Group 2 p = 0.005; vs. Group 3 p = 0.05). Adjusted RMR was significantly correlated with a number of metabolic features including WC (r = 0.32; p < 0.01), HOMA-IR (r = 0.48; p < 0.001), CRP (rho = 0.31; p = 0.02), HDL-C (r = −0.31; p = 0.02), TG (r = 0.34; p = 0.009). Adjusted RMR was not correlated with either measure of glucose metabolism (glucose or HBA1c) or blood pressure. Neither objective nor subjective habitual activity varied between groups. Regardless of whether measured by objective or subjective methods, physical activity did not display any correlations with metabolic parameters.

**Table 3:** Comparison of energy output variables across groups

**Characteristic**	**Group 1 (n = 19)**	**Group 2 (n = 16)**	**Group 3 (n = 16)**	**Group 4 (n = 18)**
**Resting metabolic rate**				
RMR (kJ.day^-1^)	4884.59 (619.37)	5232.79 (438.65)	5484.26 (625.03)	6190.93 (959.43)^a, b, c^
Adjusted RMR (kJ.day^-1^.kg^-1^)	111.7 (12.5)	112.9 (7.6)	110.5 (11.7)	122.3 (10.7) ^a, b, c^
RQ (VCO2/VO2)	0.77 (0.74-0.82)	0.75 (0.72-0.77)	0.76 (0.72-0.83)	0.79 (0.76-0.85)
**Physical activity**				
***RT3 activity monitor***				
Sedentary (min.day^-1^)	374.73 (86.55)	414.06 (104.19)	456.38 (98.89)	412.88 (103.94)
Light intensity (min.day^-1^)	390.19 (87.51)	346.31 (55.74)	346.91 (85.76)	380.26 (61.28)
Moderate intensity (min.day^-1^)	56.46 (18.0-81.4)	47.40 (17.8-76.7)	34.91 (20.1-48.6)	45.48 (28.8-54.5)
Vigorous intensity (min.day^-1^)	25.22 (3.0-37.2)	20.37 (2.1-46.1)	8.94 (0.7-9.9)	11.54 (0.4-28.8)
***Minnesota LTPAQ***				
Light AMI (MET-h.week^-1^)	0	0	0	0
Moderate AMI(MET-h.week^-1^)	14.0 (13.1)	16.7 (18.8)	12.3 (9.5)	13.7 (9.0)
Vigorous AMI (MET-h.week^-1^)	2.8 (6.4)	1.0 (2.9)	2.0 (7.6)	0.4 (6.9)
Total AMI (MET-h.week^-1^)	24.4 (17.3)	20.1 (21.3)	16.3 (15.6)	15.1 (18.8)
**Adherence to PA guidelines**	6 (32%)	5 (31%)	3 (19%)	3 (17%)

Data are presented as mean (± standard deviation) for normally distributed variables and as median (inter-quartile range) for non-normally distributed variables and as frequency (percentage) for categorical variables. Abbreviations: *RMR* resting metabolic rate, *RQ* respiratory quotient, *VCO2* carbon dioxide produced, *VO2* oxygen consumed, *LTPAQ* leisure time physical activity questionnaire, *AMI* activity metabolic index, PA physical activity.

a = significantly different from Group 1; b = significantly different from Group 2; c = significantly different from Group 3.

## Discussion

Results demonstrate that breast cancer survivors, who are both centrally obese and insulin resistant, are characterised by greater metabolic dysfunction in terms of dyslipidemia, glucose dysmetabolism, raised CRP and a higher prevalence of the MetSyn compared to women who are centrally obese in the absence of insulin resistance. This altered metabolic profile was accompanied by higher absolute and adjusted RMR levels but was not associated with differing habitual physical activity levels. These results are consistent with others which have demonstrated deteriorations in lipid-lipoprotein profile, inflammatory status and measures of glucose-insulin homeostasis when central obesity exists concurrently with insulin resistance [31,32]. In these studies, participants who were centrally obese and not insulin resistant were characterised by metabolic profiles that were similar to lean controls.

In the present study inflammatory status was measured by CRP, an acute-phase reactant secreted from the liver in response to pro-inflammatory cytokines (TNF-α and IL-6). Pro-inflammatory cytokines are secreted from the visceral adipose tissue in response to infiltration by macrophages to expanding adipose tissue and act on the liver to stimulate gluconeogenesis, resulting in increased production of hepatic glucose and consequently increased pancreatic secretion of insulin leading to the development of insulin resistance [33]. CRP levels >3 mg.L^-1^ are indicative of chronic low-grade inflammation [14]. Therefore in the current study, CRP values suggestive of low-grade inflammation (4.98 (3.46) mg.L^-1^) were observed in those who were insulin resistant. These values are comparable to those reported by Thomson and colleagues [1] who described a mean high sensitivity CRP of 5.1 (5.3) mg.L^-1^ in an overweight cohort of breast cancer survivors. Interestingly, in the present study mean CRP values did not exceed the 3 mg.L^-1^ cut-point in any of the non-insulin resistant groups. Among breast cancer survivors, significant associations have been documented between acute inflammatory markers (CRP and serum amyloid A (SAA)) and a number of anthropometric and lifestyle related prognostic indicators including BMI, increasing waist circumference and decreasing physical activity levels [34] in addition to all-cause mortality following breast cancer [5].

Chronic inflammation may also adversely influence lipid-lipoprotein profiles resulting in dyslipidemia [33]. Consequently, in the current study HDL-C was significantly lower and TG significantly higher in the insulin resistant group compared to all other groups. There is a lack of consensus regarding the presentation of dyslipidemia in obese, insulin resistant adults. Nieves and colleagues [31] reported in insulin resistant, non-obese individuals, that the presentation of dyslipidemia could be explained by differing levels of visceral fat accumulation. In contrast, Piché and colleagues [32] suggested that variations in the presentation of dyslipidemia in centrally obese adults were driven by insulin resistance. In the current study, only those who were insulin resistant showed evidence of dyslipidemia, however causality cannot be inferred.

It should be acknowledged that the HOMA-IR scores observed in this study (mean 2.09 (range: 0.47 – 6.36) were lower than observed in similar cohorts for example the HEAL study which reported mean HOMA-IR scores of 2.55 (range: 0.25 – 40.16) [3]. The HEAL study is a multicentre, multiethnic trial based in the United States of America. To the authors’ knowledge, the current study is the first to examine metabolic characteristics in an Irish cohort of breast cancer survivors. One of the limitations of this cohort is a lack of diversity variables such as BMI scores. The mean BMI of participants in the current study was 27.02 (range 19.7-39.9) kg.m^-2^ compared to 27.3 (range 16.2 – 53.3) kg.m^-2^ in those involved in HEAL [3]. While the mean values are comparable, the range and maximum values were lower in the current study. As obesity is the predominant underlying cause of insulin resistance [35], the lower HOMA-IR scores observed in the current study may be attributed to lower range of BMI values. The prevalence of obesity in Ireland is increasing steadily [36], albeit at a slower rate than observed in North America [37]. Nonetheless, the metabolic and physiological health consequences of obesity in Ireland are becoming increasingly evident and there is a need for intervention to prevent further decline.

A relationship between physical activity and metabolic markers was not observed in the current study. This is in contrast other studies in breast cancer survivors who reported correlations between physical activity and several metabolic variables including fasting insulin [28], HOMA-IR [28], CRP [34], leptin [38], IGF-I [38] and IGFBP-3 [38]. Furthermore habitual physical activity levels did not vary between groups. Sedentary behaviour was prevalent across each of these groups representing 50% (Range: 44% in Group 1 to 54% in Group 3) of waking hours. In contrast, moderate and vigorous intensity activity accounted for only 7% of waking hours (Range: 5% in Group 3 to 10% in Group 1) with less than one-third of any group achieving recommended physical activity guidelines. It could be suggested that the high percentage of sedentariness made it difficult to identify differences between equally sedentary groups in this small sample. These results, like others [7,28], represent the extent of the problem of inactivity among breast cancer survivors and may also represent the problem of inactivity at population level. Recent reports suggest that 31% of adults globally are inactive (not achieving recommended physical activity guidelines), with inactivity levels highest in Europe and America and greatest amongst women [39]. Excess sedentary behaviour is particularly concerning among breast cancer survivors however due to the association between physical inactivity and prognosis. Physical activity undertaken following breast cancer is associated with up to a 35% reduction in all-cause mortality [19,40] while inactivity has been linked to a four-fold increase in mortality risk [41].

RMR comprises the largest component of energy expenditure and therefore is an essential determinant of energy balance. Mean RMR levels (1297.53 (203.13) jK.day^-1^ (equivalent to 1297.53 (203.13) kcal.day^-1^ in the current study were comparable to those reported previously in a similar cohort (1300 (183.9) kcal.day^-1^) [1]. In addition, both absolute and adjusted RMR were significantly higher in the insulin resistant group compared to all others and demonstrated moderate-to-weak correlations with a number of metabolic variables. These results are consistent with others which report that adjusted RMR is typically increased in individuals with T2DM relative to healthy counterparts [42,43]. Weyer and colleagues [18] suggested that increases in RMR occur early in the development of T2DM in conjunction with the development of progressive metabolic abnormalities including an increase in gluconeogenesis and increased hepatic glucose productions. In contrast, there is a lack of consensus as to whether RMR is increased or decreased in individuals with clinically defined MetSyn compared to metabolically healthy counterparts [44,45]. Despite the exclusion of individuals with T2DM from the current study, results are consistent with the development of underlying glucose dysregulation. In contrast, non-insulin resistant groups with lower RMR values may be at risk of continued weight gain [46].

The aim of the current study was to identify known modifiable prognostic risk factors that may respond favourably to intervention to improve breast cancer survival. This evaluation did not compare findings to a matched control group however it is highly likely that the presented metabolic characteristics are comparable to population norms. Population statistics suggest that “normal” is characterised by obesity [37], inactivity [39] and sedentariness [47]; factors which are all associated with reduced breast cancer outcome and will contribute to metabolic decline. The challenge therefore with rehabilitation in the cancer context is the need for cancer survivors to adopt health promoting lifestyle behaviours which may be different from pre-diagnosis levels. Rehabilitation which focuses on a return to “normal” function or baseline levels is no longer adequate. Therefore it is suggested that metabolic screening to identify key modifiable risk factors and inform exercise prescription in this cohort may be more valuable than comparison to a matched control group.

## Conclusions

In recent years there has been increasing evidence of a relationship between increasing levels of obesity, and metabolic dysfunction, with poorer breast cancer prognosis [3-5,48], highlighting the importance of accurately assessing metabolic disturbance among breast cancer survivors. A greater understanding of the presentation of metabolic dysfunction will inform the design of targeted, outcome-specific weight-loss and physical activity interventions aimed at improving biomarkers of breast cancer risk. In the present study we defined the MetSyn according to the most recent consensus statement [12], and then compared metabolic profiles across groups based on increasing levels of central obesity and the presence or absence of insulin resistance. Results identify that the insulin resistant group displayed alterations in both measures of insulin-glucose homeostasis and CRP, two significant predictors of breast cancer outcome [3,5]. In addition, results show that increasing central obesity in the absence of insulin resistance was not associated with a greater deterioration in metabolic profile when compared to lean controls, suggesting that the assessment of insulin resistance is crucial in the assessment of metabolic dysfunction for identifying potentially prognostic characteristics. While physical activity was not associated with differing levels of metabolic dysfunction, the high prevalence of sedentary time and low adherence to physical activity guidelines is concerning. Elevated RMR values in the insulin resistant group however are consistent with others which have shown that RMR is increased in the presence of serious metabolic dysfunction and glucose dysregulation. In breast cancer survivors, a comprehensive assessment of metabolic dysfunction is required to identify profiles that may potentially have greater prognostic significance and may inform the design of future targeted exercise interventions.

## Competing interests

The authors declare that they have no competing interests.

## Authors’ contributions

All authors (EG, EC, MJK and JH) developed the idea for this study. Measurements and data analysis was completed by EG. EC and MJK provided medical advice regarding interpretation of the data. EG was responsible for drafting the manuscript with contributions from JH. All authors approved the final version of the manuscript.
